# *Wolffia globosa–Mankai* Plant-Based Protein Contains Bioactive Vitamin B_12_ and Is Well Absorbed in Humans

**DOI:** 10.3390/nu12103067

**Published:** 2020-10-08

**Authors:** Ilan Sela, Anat Yaskolka Meir, Alexander Brandis, Rosa Krajmalnik-Brown, Lydia Zeibich, Debbie Chang, Blake Dirks, Gal Tsaban, Alon Kaplan, Ehud Rinott, Hila Zelicha, Shira Arinos, Uta Ceglarek, Berend Isermann, Miri Lapidot, Ralph Green, Iris Shai

**Affiliations:** 1 Research and Development Department, Hinoman Ltd., Rishon Lezion 7546302, Israel; ilansal@gmail.com (I.S.); shirabella@gmail.com (S.A.); miri@hinoman.com (M.L.); 2 Faculty of Health Sciences, Ben-Gurion University of the Negev, Beer-Sheva 8410501, Israel; anatyas@post.bgu.ac.il (A.Y.M.); gtsaban@gmail.com (G.T.); alonkaplan47@gmail.com (A.K.); ehudrinott@gmail.com (E.R.); hila.zelicha@gmail.com (H.Z.); 3 Targeted Metabolomics Unit, Life Sciences Core Facilities Weizmann Institute of Science, Rehovot 76100, Israel; Alexander.Brandis@weizmann.ac.il; 4 School of Sustainable Engineering and the Built Environment, Biodesign Center for Health Through Microbiomes, Arizona State University, Tempe, AZ 85281, USA; Dr.Rosy@asu.edu; 5 Biodesign Swette Center for Environmental Biotechnology, Arizona State University, Tempe, AZ 85287, USA; lzeibich@asu.edu (L.Z.); dcchang@asu.edu (D.C.); bedirks@asu.edu (B.D.); 6 Institute for Laboratory Medicine, University of Leipzig Medical Center, 04103 Leipzig, Germany; Uta.Ceglarek@medizin.uni-leipzig.de (U.C.); berend.isermann@medizin.uni-leipzig.de (B.I.); 7 Department of Pathology and Laboratory Medicine, University of California Davis School of Medicine, Sacramento, CA 95817, USA; 8 Department of Nutrition, Harvard T.H. Chan School of Public Health, Boston, MA 02115, USA

**Keywords:** *Wolffia globosa*, vitamin B_12_, plant-based nutrition, flexitarians, weight loss, sustainability

## Abstract

Background: Rare plants that contain corrinoid compounds mostly comprise cobalamin analogues, which may compete with cobalamin (vitamin B_12_ (B_12_)) metabolism. We examined the presence of B_12_ in a cultivated strain of an aquatic plant: *Wolffia globosa* (Mankai), and predicted functional pathways using gut-bioreactor, and the effects of long-term Mankai consumption as a partial meat substitute, on serum B_12_ concentrations. Methods: We used microbiological assay, liquid-chromatography/electrospray-ionization-tandem-mass-spectrometry (LC-MS/MS), and anoxic bioreactors for the B_12_ experiments. We explored the effect of a green Mediterranean/low-meat diet, containing 100 g of frozen Mankai shake/day, on serum B_12_ levels during the 18-month DIRECT-PLUS (ID:NCT03020186) weight-loss trial, compared with control and Mediterranean diet groups. Results: The B_12_ content of Mankai was consistent at different seasons (*p* = 0.76). Several cobalamin congeners (Hydroxocobalamin(OH-B_12_); 5-deoxyadenosylcobalamin(Ado-B_12_); methylcobalamin(Me-B_12_); cyanocobalamin(CN-B_12_)) were identified in Mankai extracts, whereas no pseudo B_12_ was detected. A higher abundance of 16S-rRNA gene amplicon sequences associated with a genome containing a KEGG ortholog involved in microbial B_12_ metabolism were observed, compared with control bioreactors that lacked Mankai. Following the DIRECT-PLUS intervention (*n* = 294 participants; retention-rate = 89%; baseline B_12_ = 420.5 ± 187.8 pg/mL), serum B_12_ increased by 5.2% in control, 9.9% in Mediterranean, and 15.4% in Mankai-containing green Mediterranean/low-meat diets (*p* = 0.025 between extreme groups). Conclusions: Mankai plant contains bioactive B_12_ compounds and could serve as a B_12_ plant-based food source.

## 1. Introduction

Cobalamin is an essential nutrient for humans. It has the largest molecular mass (1355.4 g/mol) and the most complex structure of all vitamins [1]. The term “vitamin B_12_” is the name usually used for cyanocobalamin (CN-B_12_), which is the most chemically stable form of cobalamin. In this study, vitamin B_12_ will be used to refer to all corrinoids exhibiting the qualitative biological activity of CN-B_12_ [2], including the following three natural forms: Hydroxocobalamin (OH-B_12_), 5-deoxyadenosylcobalamin (Ado-B_12_), and methylcobalamin (Me-B_12_). CN-B_12_ is the form used in most dietary supplements and is readily converted to the coenzyme forms, Me-B_12_ and Ado-B_12_ in the body [1]. Me-B_12_ functions as a cofactor for the methionine synthase reaction involved in the conversion of homocysteine to methionine through a transfer of a methyl group from methyltetrahydrofolate; Ado-B_12_ functions as a cofactor for methylmalonyl-CoA mutase in which methylmalonyl-CoA, a product of amino acid and odd-chain fatty acid catabolism, is converted to succinyl-CoA [1]. At the cellular level, these enzymes play an important role in several crucial functions, such as DNA synthesis, methylation, and mitochondrial metabolism [3,4].

De novo synthesis of vitamin B_12_ appears to be restricted to some bacteria and archaea [2,5]. The vitamin is therefore found solely in foods fermented by B_12_-producing bacteria, or in those derived from the tissues of animals that have ingested B_12_-containing foods or which have obtained it from B_12_-producing microbiota of their commensal microflora [2]. Hence, animal-derived foods (meat, milk, eggs, and shellfish) are considered to be the exclusive dietary source of B_12_ vitamin in humans [5,6]. However, a preference for diets that limit intake of animal products has arisen during the past decade, largely from the belief that lower animal-source protein diets reduce risk factors for cardiometabolic diseases, such as hypertension, dyslipidemia, hyperglycemia, type 2 diabetes, and cardiovascular diseases [7,8,9,10]. On the other hand, since vitamin B_12_ is not measurably present in plant-based foods, individuals adhering to a vegan diet without vitamin B_12_ supplementation are at risk of developing vitamin B_12_ deficiency with potentially serious and sometimes irreversible consequences [3,11]. Indeed, various types of edible algae have been reported to contain vitamin B_12_ [4,12]. However, recent data indicate that pseudo B_12_ forms, such as OH-pseudoB_12_, Ado-pseudoB_12_, Me-pseudoB_12_, and CN-pseudoB_12_, which are considered inactive in humans, and might compete with B_12_, are the predominant corrinoids present in the algae [4,12].

*Wolffia globosa* ‘Mankai’ is an aquatic plant of the duckweed family recently identified for its nutritional value [13,14]. It has a unique nutritional composition profile, which includes about 45% protein of its dry weight, with all nine essential amino acids in a ratio equivalent to that of egg protein [15], a source of omega-3 fatty acids [16]; dietary fiber; polyphenols; iron; and several other micronutrients that tend to have low abundance in animal-based foods diets (e.g., vitamin A as beta-carotene, riboflavin, vitamin B_6,_ and folate). One cup of Mankai shake, which is equivalent to ~20 g of dry matter, provides the following proportions of recommended intakes: 18% whole bioavailable protein [15], 75% bioavailable iron [17], 60% folic acid, and 21% vitamin B_12_. In our previous bioavailability study, we found, unexpectedly, that the serum vitamin B_12_ concentrations increase and attain higher levels than the increase observed following other protein source meals [15].

To exclude the sporadic presence of B_12_ and to evaluate the stability levels in Mankai biomass, various Mankai samples, grown under different conditions, ranging from lab scale under artificial light to commercial scale under sunlight, were examined for their B_12_ content by two different methods. In the DIRECT PLUS weight-loss trial, among 294 participants with abdominal obesity and normal B_12_ levels, we explored the effect of an 18-month intake of Mankai, consumed as an evening green shake, as a partial protein plant-based substitute, on vitamin B_12_ serum levels. Besides, we examined changes in the gut microbiome when directly exposed to Mankai using anoxic bioreactors, to simulate the human colon environment/microbiota. We hypothesized that Mankai might serve as a consistent vitamin B_12_ source, despite the reduction in red meat intake.

## 2. Materials and Methods

### 2.1. Mankai Laboratory Analyses

#### 2.1.1. Plant Sources

##### Vitamin B_12_ Detection in Plants Cultivated Under Greenhouse Conditions

Cultivated plant samples: Mankai biomass is grown in closed controlled highly monitored aquatic greenhouses using a proprietary precision agriculture cultivation system. We sampled the plant for B_12_ analysis at different seasons during the years 2014 to 2019. Plant biomass was sieve harvested, washed with tap water for 2 min, and dried in a food dehydrator (Excalibur, Sacramento, USA) at 65 °C for 16 h. Each dried plant sample was stored in a vacuum-sealed aluminum bag at 4 °C, until analysis was performed.

##### Vitamin B_12_ Detection in Axenic Culture

Generating axenic culture: Plant sterilization was achieved by submerging and agitating plants in predetermined concentrations of sodium hypochlorite for 1–3 min. Treated fronds were transferred to a 12-well plate containing sterile Hoagland solution (MgSO_4_·7H_2_O 0.246 g/L, Ca(NO_3_)_2_·4H_2_O 542 mg/L, KH_2_PO_4_ 68 mg/L, KNO_3_ 250 mg/L, FeNa·EDTA 37 mg/L, H_3_BO_3_ 1.5 mg/L, MnCl_2_·4H_2_O 9.1 mg/L, ZnSO_4_·7H_2_O 0.11 mg/L, Na_2_MoO_4_·2H_2_O 0.045 mg/L, CuSO_4_·5H_2_O 0.045 mg/L, and 1% Sucrose (All purchased from Fisher Scientific, Leicestershire, UK). The Hoagland formulation does not contain cobalt compounds. Furthermore, ICP-MS analysis performed by an accredited laboratory was applied to this 10× concentrated Hoagland solution and revealed no cobalt traces (<0.01 ppm). The plate was covered with aluminum foil and kept at 25 °C for 24 h. After the foil was removed, the plants were allowed to recover for an additional 6 days under a 24-h light regime at 120 µE. Bleached mother fronds with green daughter fronds were transferred to a new sterile well to establish a sterile Mankai culture. Three sterile cultures, derived from three independent treatments, were continuously grown in vitamin B_12_-free Hoagland medium that was replaced once a week. Culture sterility was verified by incubation of whole and crushed fronds on PCA (plate count agar, Neogen, Michigan, USA) at 30 °C for at least 5 days. Vitamin B_12_ analysis was performed on 5-month-old independent plant cultures that were intensively washed with running tap water for two minutes and dried in a food dehydrator as described above.

#### 2.1.2. Vitamin B_12_ Analyses

##### Bioassay Method

Total vitamin B_12_ in the plant samples was measured by the AOAC 952.20 microbiological analytical method, utilizing the B_12_-requiring bacterium *Lactobacillus delbrueckii* subsp. lactis ATCC7830, which is the established vitamin B_12_ determination method for foods [18]. The analysis was performed by Eurofins Laboratories, Inc. (Des Moines, IA, USA) and by Bactochem Ltd. (Nes Ziona, Israel). Some tests were done by Hinoman Ltd., analyzing one gram of dried plant by the Vitafast B_12_ microbiological assay kit (R-Biopharm, AG, Darmstadt, Germany) according to the manufacturer’s instructions.

##### Liquid Chromatography/Electrospray Ionization Tandem Mass Spectrometry (ESI LC-MS/MS) Assay

Extraction of Vitamin B_12_: The extraction of dried Mankai samples and two commercial Spirulina powders that served as a reference for pseudo vitamin B_12_ are described in Supplementary File S1.

Purification of vitamin B_12_ and LC-MS/MS: B_12_ extracts were evaporated to dryness under reduced pressure and then re-dissolved in 9 mL of double-distilled water. The obtained solutions were loaded onto an immunoaffinity column (EASI-EXTRACT vitamin B_12_ immunoaffinity column (AOAC 2014.02), R-Biopharm AG, Darmstadt, Germany) and purified according to the manufacturer’s protocol. The recovery efficiency of pseudo CN-B_12_ was considered to be similar to that of authentic CN-B_12_. Subsequently, 10-μL aliquots of extracts were analyzed in optimized conditions determined using individual B_12_ standards. The concentrations based on standard curves were calculated using TargetLynx (Waters, Milford, MA, USA). The LC-MS/MS assay was performed at the Life Sciences Core Facilities of Weizmann Institute of Science. Further extraction and purification methods, as well as retention times and Multiple Reaction Monitoring (MRM) parameters for the detection of corrinoids, are given in Supplementary File S1 and Table S1.

### 2.2. The DIRECT PLUS Dietary Intervention Trial

#### 2.2.1. Study Design

The 18-month DIRECT-PLUS (dietary intervention randomized controlled trial polyphenols-unprocessed) trial (clinicaltrials.gov ID: NCT03020186) aimed to address the residual beneficial effect of a green Mediterranean diet, richer in green plants and lower in meat, compared with other healthy lifestyle strategies. The trial was initiated in May 2017 and was conducted in an isolated workplace (Nuclear Research Center Negev (NRCN), Dimona, Israel), where a monitored lunch was provided. This workplace includes a medical department where most of the medical measurements were taken and where lifestyle intervention sessions were held. Of the 378 volunteers, 294 met the inclusion criteria of age >30 years and characterized by abdominal obesity (waist circumference (WC): men > 102 cm, women > 88 cm) or dyslipidemia (TG > 150 mg/dL and high-density lipoprotein cholesterol (HDL-c) ≤ 40 mg/dL for men, ≤ 50 mg/dL for women). Exclusion criteria are detailed in Supplementary File S2.

All subjects gave their informed consent for inclusion before they participated in the study. The study was conducted in accordance with the Declaration of Helsinki, and the protocol was approved by the Medical Ethics Board and Institutional Review Board at Soroka University Medical Centre, Be’er Sheva, Israel (0280-16-SOR). All participants did not receive any financial compensation.

#### 2.2.2. Randomization and Intervention

Randomization and intervention were described elsewhere [17,19]. Briefly, participants were randomly assigned to one of three intervention groups, all combined with physical activity recommendation (along with a free gym membership):

Healthy dietary guidelines (HDG) group: In addition to the workout program, the participants received basic health-promoting guidelines for achieving a healthy diet.

Mediterranean (MED) group: In addition to the workout program, participants were instructed to adopt a calorie-restricted Mediterranean diet as described in our previous trials: DIRECT [20] and CENTRAL [21] trials, supplemented with 28 g/day of walnuts.

Green Mediterranean (green-MED) group: In addition to the Mediterranean intervention (including the provided walnuts), the green Mediterranean dieters were further guided to avoid red and processed meat, with the diet being richer in plants and polyphenols. The participants were guided to further consume the two following provided items: 3–4 cups/day of 100 g frozen cubes of Mankai (whole plant), replacing dinner and a potential source of protein, iron, and vitamin B_12_. The MED and green-MED diets were equally calorie restricted (1500–1800 kcal/day for men and 1200–1400 kcal/day for women). All the above (walnuts, green tea, and Mankai) were provided free of charge.

#### 2.2.3. Outcomes

Blood samples were taken at 8:00 AM after a 12-h fast, at baseline and after 18 months of intervention. The samples were centrifuged and stored at −80 °C. Serum vitamin B_12_ was analyzed with a competitive Elektro Chemiluminescence-Immuno Assay “ECLIA” (Cobas 8000, Roche Diagnostics, Mannheim, Germany) using Intrinsic Factor as a binding protein. Serum folate was also measured by the ECLIA competitive approach and was used as a marker for green leaf consumption [22]. All biochemical analyses were performed at the laboratories of the University of Leipzig, Germany. Chemical and hematological parameters in freshly drawn blood samples were assessed at the workplace clinic at baseline and at the end of the intervention measurements (±1 month before/after initiating blood draws). Additional outcomes measures (i.e., anthropometric, electronic questionnaires) are presented in Supplementary File S3.

#### 2.2.4. Statistical Analysis

The primary outcomes of the DIRECT PLUS study, as stated in clinicaltrials.gov, were 18-month changes in adiposity parameters (a flow diagram for the study is presented in Figure S1). In this analysis, we primarily aimed to assess serum vitamin B_12_ change during the study period. Continuous variables are presented as means ± standard deviations for normally distributed variables and medians for non-normally distributed variables, with the Kolmogorov–Smirnov test used to determine the variable’s distribution. Nominal variables are expressed as numbers and percentages. Differences between time points were tested using the paired sample *T*-test or Wilcoxon test. Differences between groups (i.e., intervention groups or tertiles) were tested using analysis of variance (ANOVA), Kruskal–Wallis test, or Chi-square test. Ln transformations were applied when necessary to achieve normal distribution. Kendal Tau correlation was used to examine *p* of trend. Multiple comparisons were addressed using the Tukey post hoc test (for ANOVA), and Bonferroni correction (for Kruskal–Wallis). For adjustments, we used general linear regression models, with the specific adjustments detailed with the results. Sample size calculations were detailed elsewhere [17]. Statistical analysis was performed using SPSS (version 25.0, IBM, Armonk, NY, USA). Statistical significance was set at 0.05 level, two-sided.

### 2.3. Anoxic Gut Microbiome Bioreactors Pilot Experiment

#### 2.3.1. Microbiota Reactors (Human Fecal Mixture)

A mixture of human fecal samples obtained from 20 healthy male and female volunteers (age: 18–65 years) collected for a research study in 2017 (Krajmalnik-Brown Lab; IRB#STUDY00004850, Arizona State University) was used to inoculate anoxic bioreactors. After donation, fecal samples were kept at 4 °C and 1 g of sample was supplemented with 500 μL of 40% (*v*/*v*) anaerobic glycerol solution. The fecal mixtures, consisting of 20 homogenized fecal samples obtained from each donor, were stored in anaerobic freezer bags at −80 °C. Prior to use, 1 mL of fecal mixture was added to a serum bottle filled with 70 mL of anoxic Base medium (see below). The bottle, containing the starter culture, was placed in a shaking incubator for 24 h at 100 rpm and 37 °C. Headspace gas quantification was used to confirm microbial activity.

#### 2.3.2. Media, Anoxic Bioreactor, Mankai Lysate, and Sampling

Two anoxic media were used to examine the potential effect of Mankai on human-derived gut microbiota. Both media were based on the protocol described by McDonald et al. [23], with the following modification to provide the same chemical oxygen demand (COD) amount (200 meq/L) to all treatments. The final media consisted of an anoxic micronutrient-containing solution and an anoxic macronutrient solution (Table S2). COD was measured to quantify the reducing equivalents in both solutions. To obtain a (a) base medium for the bioreactors that lacked Mankai and for the starter culture (see above), and (b) Mankai medium for the Mankai-supplemented bioreactors, micronutrient-containing solution, and macronutrient solution were combined, accordingly (Table S2). Before bioreactor inoculation (adding 1 mL of the starter culture (see above)), Mankai lysate was prepared by blending 5 g of frozen Mankai biomass (*Wolffia globosa* ‘Mankai’) with 400 mL of deionized (DI) water for 5 min and subsequently flushing with nitrogen for 5 min. After inoculation and before the first fill and draw, the bioreactors were incubated for 48 h in the dark at 37 °C and mixed continuously at 100 rpm. Full details regarding the media, anoxic bioreactor, Mankai lysate, and the sampling are provided in Supplementary File S4.

#### 2.3.3. Chemical and Molecular Analysis

Total COD was determined by adding 400 µL of solution, medium, or lysate to a HACH COD vial (HACH, High Range 20–1500 mg COD/L) with 1600 μL of DI water followed by a 2-h incubation at 150 °C (HACH DRB200). The vials were then cooled and measured for COD concentration in mgCOD/L using a spectrophotometer (HACH DR2800 Laboratory Spectrophotometer). For microbiome composition analysis, we performed 16S rRNA gene amplicon sequencing using Illumina sequencing technology and found core differences as described [24,25]. Further detailing regarding the 16S rRNA amplicon sequences is presented in Supplementary File S5.

## 3. Results

### 3.1. Mankai Plant Analyses

#### 3.1.1. Content and Stability of Vitamin B_12_ Levels during Different Seasons

**Figure 1:**
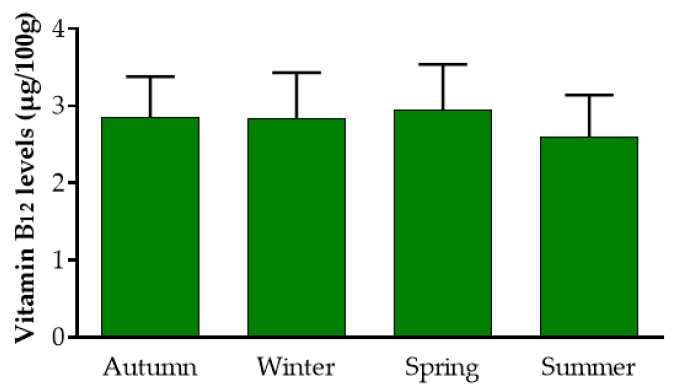
Stability of vitamin B_12_ levels in Mankai™ along the year. “Autumn” refers to water temperatures of 22–24.5 °C and 10:20–10:50 h of light. “Winter” refers to water temperatures of 17–20 °C and 10–10:20 h of light. “Spring” refers to water temperatures of 21–24 °C and 11:30–13:30 h of light. “Summer” refers to water temperatures of 25–29 °C and 13:50–14:15 h of light. For each season, the weekly average water temperatures and daily light hours relate to the sampling date.

Overall, Mankai contained 2.8 ± 0.5 µg B_12_/100 g dry weight (DW) and the concentration remained relatively stable during the seasons (Figure 1), regardless of the water temperature (17 °C–29 °C) or duration of light hours (10–14): autumn: 2.84 ± 0.5 µg/100 g DW, *n* = 5 (range: 2.34 µg/100 g to 3.62 µg/100 g DW); winter: 2.83 ± 0.6 µg/100 g DW, *n* = 5 (range: 1.96 µg/100 g to 3.44 µg/100 g DW); spring: 2.94 ± 0.6 µg/100 g DW, *n* = 4 (range: 2.19 µg /100 g to 3.52 µg/100 g DW); and summer: 2.6 ± 0.5 µg/100 g DW, *n* = 6 (range: 1.83 µg/100 g to 3.26 µg/100 g DW). (*p* = 0.76 between seasons).

#### 3.1.2. Inherent Presence of Vitamin B_12_ in Mankai Axenic Cultures

B_12_ concentrations in three independent axenic cultures, which were vegetatively propagated for at least 5 months post establishment, were 2.08, 2.34, and 1.6 µg/100 g DW.

#### 3.1.3. Identification of Vitamin B12 Purified from Mankai

**Figure 2:**
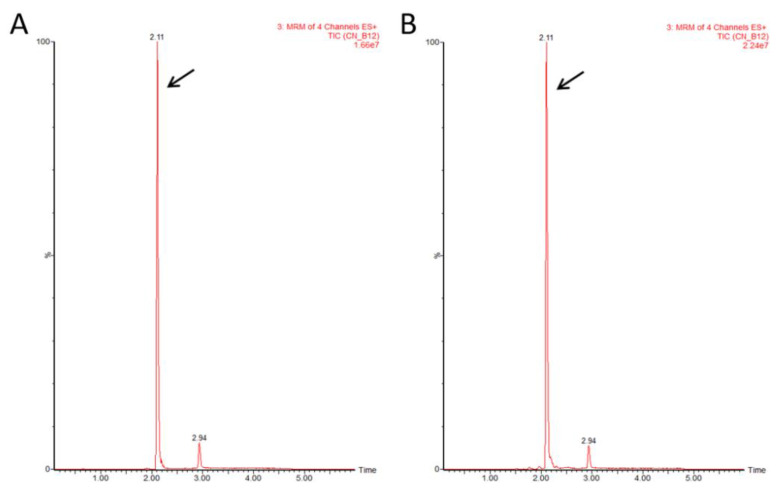
Liquid chromatography/electrospray ionization tandem mass spectrometry (LC-MS/MS) chromatograms of CN-B_12_. (**A**) Retention time for CN-B_12_ standard (arrow). (**B**) Retention time for CN-B_12_ extracted from Mankai sample (arrow). ES, electrospray; MRM, multiple reaction monitoring; TIC, total ion current.

To verify that the corrinoid detected by the bioassay was indeed a bioactive form of cobalamin, we used LC-MS/MS. The presence of the active form was validated in all 10 tested samples: four plant samples representing three different seasons (spring, summer, and autumn) and 6 samples grown under indoor conditions. Representative data of a Mankai sample collected during mid-March 2019 from an outdoor basin are shown in Figure 2. Standard CN-B_12_ was eluted as a peak with a retention time of 2.11 min (Figure 2A) and the plant extract sample showed a corresponding peak with the same retention time (Figure 2B) for all MRM transitions. The intensity ratios between individual MRM signals were kept similar in both standard and plant samples (Figure S2).

#### 3.1.4. Quantification of Total Vitamin B_12_ Purified from Mankai

The extractions described above were performed in the presence of KCN, which converts the naturally occurring forms of cobalamin to the stable CN-B_12_ form. Since this conversion is not always complete [26], we analyzed all four vitamin B_12_ forms by LC-MS/MS, with the aim of determining the total vitamin B_12_ content of Mankai. Commercial OH-B_12_, CN-B_12_, Ado-B_12_, and Me-B_12_ standards were eluted as peaks with retention times of 1.87, 2.1, 2.25, and 2.31 min, respectively, and the plant extract samples showed corresponding peaks with the same retention times (Figure S3). The intensity ratios between individual MRM signals were kept similar in both standard and plant samples (data not shown). These results indicate that all three natural forms were present in Mankai and that incomplete conversion to CN-B_12_ had occurred. The identification of CN-B_12_, OH-B_12_, Ado-B_12_, and Me-B_12_ was further validated by four, three, two, and four MRMs, respectively. In order to calculate the total vitamin B_12_ in the plants, we measured the recovery rate of each form by analyzing the standards, with or without immunoaffinity column purification. Namely, the solutions containing the standard mix of four B_12_ forms in equal amounts were divided in two halves. One half was diluted with acetate buffer and passed through a EASI-EXTRACT vitamin B_12_ immunoaffinity column according to the manufacturer’s purification protocol. The obtained eluate was evaporated and re-dissolved to the same volume as the second half. Samples thus obtained were analyzed by LC-MS/MS. The results showed recovery rates of 55%, 37%, 16%, and 100% for CN-B_12_, OH-B_12_, Ado-B_12_, and Me-B_12_, respectively. The analysis was performed on three plant samples that were obtained from greenhouse cultivation basins during spring, summer, and autumn. The amount of each form was then measured in plant extracts and the total B_12_ level was calculated according to the recovery rates. The data showed that the average total authentic vitamin B_12_ concentrations in Mankai is 3.23 µg ± 0.5/100 g DW and stable during different seasons: spring 2.86 µg, summer 3.84 µg, and autumn 2.99 µg/100 g DW. These concentrations are in line with the results received by the bioassay method.

#### 3.1.5. Authentic CN-B_12_ and Pseudo CN-B_12_ in Mankai

**Figure 3:**
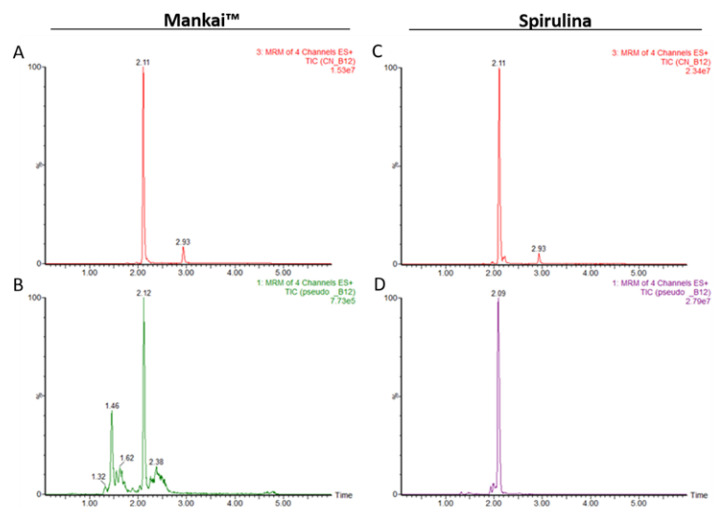
A comparison of chromatograms of TIC for authentic CN-B_12_ and pseudo CN-B_12_ in Mankai and spirulina samples. (**A**–**C**): Active CN-B_12_; (**B**–**D**) Pseudo CN-B_12_ in Mankai™ (**A**,**B**) and spirulina (**C**,**D**) samples. In panel B, a peak at 2.12 min does not represent pseudo CN-B_12_ because pseudo CN-B_12_ should appear before the peak of CN-B_12_ [27,28] as is observed with a peak from a spirulina sample (at 2.09 min, panel D) and is present not just in one but in all 4 MRM transitions at measurable levels (Figure S4). ES, electrospray; MRM, multiple reaction monitoring; TIC, total ion current.

To further study Mankai as a vitamin B_12_ food source, we estimated the concentrations of pseudo B_12_ in the plant. To this end, we used LC-MS/MS to analyze samples of spirulina that are known to produce large amounts of pseudo B_12_ [27] and therefore can be used as a reference. This measurement was performed assuming similar ionization products for both CN-B_12_ and pseudo CN-B_12_, so the standard CN-B_12_ curve was used as a reference to quantify both compounds. Based on the different molecular masses of CN-B_12_ and pseudo CN-B_12_, the data revealed the presence of CN-B_12_ and pseudo CN-B_12_ in a ratio 1:3 in two different spirulina samples, whereas no pseudo CN-B_12_ was detected in the Mankai samples (Figure 3 and Figure S4).

### 3.2. DIRECT PLUS Trial

#### 3.2.1. Baseline Characteristics

The baseline characteristics are presented in Table 1. The mean vitamin B_12_ concentration was 420.4 ± 187.8 pg/mL (range: 150–1500 pg/mL), with a mean of 414.3 ± 182.5 pg/mL for men and 465.5 ± 220.9 pg/mL for women (*p* = 0.21 between sexes). Triglyceride levels were lower in the highest vitamin B_12_ tertile compared with the lowest tertile (*p* = 0.01). Details regarding baseline vitamin supplementation are presented in Supplementary File S6.

All chemical and hematological parameters (mean corpuscular volume (MCV), mean cell hemoglobin (MCH), mean corpuscular hemoglobin concentration (MCHC), red blood cells (RBCs) hemoglobin and hematocrit; *n* = 290 for hemoglobin; *n* = 124 for other parameters) were similar and within the normal range across intervention groups (data not shown).

#### 3.2.2. The Effect of the Intervention on Serum B_12_ Levels

The trial’s 18-month subject retention rate was 89.8%. Higher and similar weight reductions were observed, following a caloric deficit, in the two MED groups (MED: −2.9 ± 5.2%; Green-MED/low-meat: −3.9 ± 6.5%) compared with the HDG group (−0.6 ± 5.1%, *p* < 0.05 for both MEDs vs. HDG). Overall, the green-MED/low-meat diet group significantly increased intake of fish, Mankai, and green tea, and decreased red meat and poultry compared with the two other groups (*p* < 0.01 for all). Both MED groups increased egg and milk consumption compared with the HDG group [16]. Vitamin supplementation usage at the end of the intervention did not differ between the intervention groups (Supplementary File S6).

**Figure 4:**
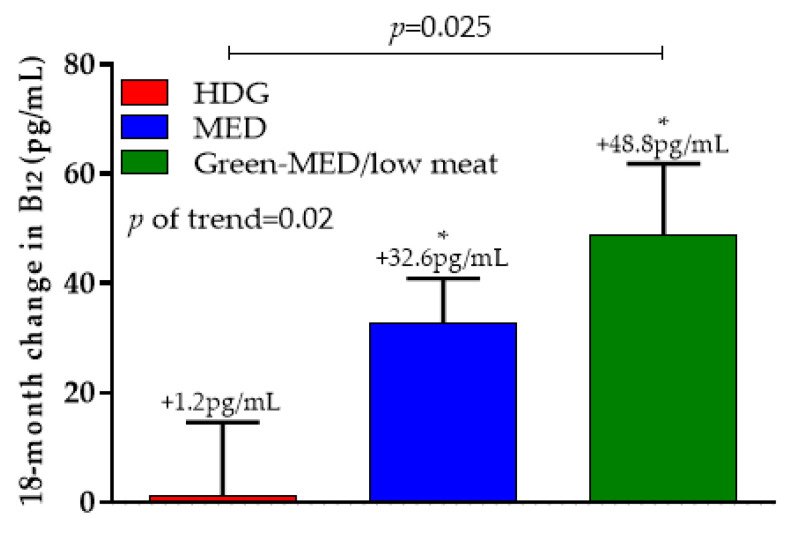
The 18-month change in serum vitamin B_12_ across intervention groups. * Indicates within-group change (baseline vs. T18) at the 0.05 level. Data presented as means and SEM. HDG, healthy dietary guidelines; MED, Mediterranean.

Differences in serum vitamin B_12_ concentrations between intervention groups are presented in Figure 4. After 18 months, the HDG group had a non-significant 1.245 ± 126.5 pg/mL (+5.2%) change in serum vitamin B_12_ levels (*p* = 0.93 vs. baseline), while MED had a significant increase in serum vitamin B_12_ levels (32.6 ± 76.2 pg/mL (+9.9%); *p* < 0.001 vs. baseline) as well in group Green-MED/low-meat (48.8 ± 124.9 pg/mL (+15.4%); *p* < 0.001 vs. baseline). *P*-of-trend was observed between the groups (*p* = 0.02), with a significant difference between the HDG and the green-MED/low-meat groups (*p* = 0.025). When further adjusted for age, sex, and baseline B_12_ concentrations, these significant differences remained (*p* = 0.01). 

#### 3.2.3. Changes in chemical and hematological Parameters

After 18 months of intervention, among the sub-group of participants with available hematological and chemical measurements (*n* = 71 for hemoglobin; *n* = 41 for other hematological parameters), all groups demonstrated no significant changes in MCV, MCH, MCHC, RBC hemoglobin, or hematocrit, and also did not differ between the groups (*p* > 0.05 for all comparisons). 

#### 3.2.4. Dietary Vitamin B_12_ Sources

**Figure 5:**
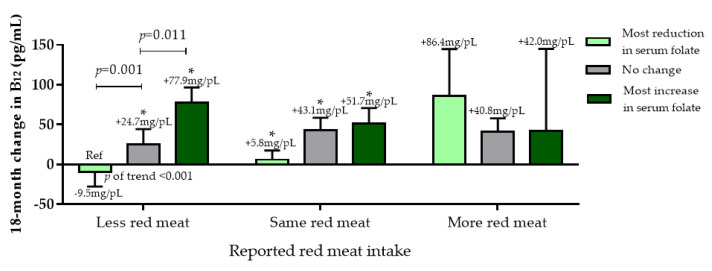
Red meat consumption change at the end of the intervention (tertiles) vs. 18-month serum folate change (tertiles) vs. 18-month change in vitamin B_12._ * indicated within-group significance (baseline vs. T18) at the 0.05 level. Data presented as means and SEM.

Next, we examined red meat (reported as increased, decreased, or no change in consumption) vs. Mankai frequency of intake tertiles, and change in serum folate (Green-MED/low-meat group only). Those who decreased red meat intake throughout the intervention showed a significantly increased serum folate associated with more frequent intake of Mankai (*p* of trend < 0.05; Figure S5a). Across all intervention groups, among those who decreased red meat consumption, increased serum folate was associated with increased serum vitamin B_12_ (*p* < 0.05) (Figure 5). The less red meat/increased serum folate group had a comparable increase of serum vitamin B_12_ to the mor -red meat/decreased serum folate group (86.0 ± 117.6 pg/mL vs. 77.9 ± 118.6 pg/mL, *p* = 0.88). In a similar analysis, replacing red meat with fish, we observed that among participants who increased fish intake throughout the intervention, an increase in vitamin B_12_ was observed, as well as serum folate (*p* of trend <0.01 for both). Significant increases in serum folate and vitamin B_12_ were observed for participants who both consumed more fish and Mankai, and demonstrated an increase in serum folate levels, as compared with other groups (Figure S5b,c).

No significant difference between extreme groups less red meat/most increase in serum folate and more red meat/most reduction in serum folate was observed.

### 3.3. Anoxic Bioreactors Pilot Experiment

#### Predicted Functional Pathways-Gut Bioreactor

Based on 16S rRNA gene amplicon sequences obtained from all bioreactors at the end of incubation (day 14), we predicted KEGG (Kyoto Encyclopedia of Genes and Genomes) present in the genomes of the bacteria identified, using Predicted functional profile analysis via PICRUSt [29]. This analysis, allowing us to predict KEGGS and the linear discriminant analysis effect size (LEFSE), showed that Mankai-supplemented bioreactors displayed a significantly higher relative abundance of 16S rRNA gene sequences associated with a genome containing a KEGG ortholog involved in vitamin B_12_ uptake (*btuB*; KEGG identifier K16092) than control bioreactors that lacked Mankai. Statistical analyses revealed a linear discriminant analysis (LDA) score of 2.19 (log10) and a relative *btuB* abundance of 0.034 ± 0.008 and 0.00 ± 0.001 in Mankai-supplemented reactors and reactors that lacked Mankai, respectively (*p* < 0.05 between reactors).

In total, 1180 of 5257 different 16S rRNA gene amplicon sequences were identified in the three replicated Mankai-supplemented bioreactors that contributed to the increased predicted abundance of microbes containing btuB. Six 16S rRNA gene amplicon sequences displayed a greater than 0.5% relative 16S rRNA gene amplicon abundance, and three of these sequences (closely related to *Aeromonas hydrophila*, *Pelomonas aquatica*, and *Geobacter anodireducens*) were present in all three replicated Mankai-supplemented bioreactors (Figure S3). In marked contrast, only nine different 16S rRNA gene amplicon sequences—associated with microbes potentially containing btuB—were identified in the control reactors that lacked Mankai, of which (a) five of these nine were present in all three replicates and (b) only one sequence (closely related to *Escherichia coli*) displayed a relative abundance greater than 0.5% (Table S3).

## 4. Discussion

In the current study, we examined, using different methodologies, the presence of vitamin B_12_ in a cultivated strain of *Wolffia globosa* (Mankai). We found that Mankai, cultured under closed-controlled greenhouse conditions, contains a substantial amount of the known bioactive forms of vitamin B_12_ and that its presence is stable throughout the year. In inoculated gut microbiome anoxic bioreactors, a significantly higher relative abundance of 16S rRNA gene sequences associated with a genome containing the KEGG ortholog involved in vitamin B_12_ uptake was observed, compared with control bioreactors that lacked Mankai. In our human studies, results suggest that long-term consumption of this plant, as part of a whole flexitarian diet, may increase rather than impair vitamin B_12_ levels, without additional red meat intake. To our knowledge, this is the first reported study on the B_12_ content and bioavailability in duckweed and specifically in *Wolffia globosa*.

Although some evidence for the presence of vitamin B_12_ in Actinorhizal plants has been reported [28], it is generally recognized that vitamin B_12_ is absent from plant-derived food sources [1,2,5,30]. Plants neither require nor synthesize vitamin B_12_ because they contain no cobalamin-dependent enzymes and instead encode a B_12_-independent form of methionine synthase [31]. To carefully examine our hypothesis regarding the presence of vitamin B_12_ in Mankai, we analyzed, over a period of 5 years, samples that were obtained from intensively grown plant cultures. Repeated microbiological assay analyses revealed the presence of stable levels of vitamin B_12_ in Mankai. Furthermore, to exclude B_12_ presence due to absorption from an external source, we tested vitamin B_12_ in axenic Mankai cultures, generated by propagating a green daughter frond that emerged from a bleached mother frond, for several months under sterile conditions. We speculated that in Mankai plants grown under these conditions, the level of any absorbed vitamin B_12_ from an external source, such as occasional bacteria or microalgal contamination, would be expected to decline and probably become undetectable in the axenic culture as the plants propagated for successive generations in the sterile culture and as the biomass increased by several orders. However, B_12_ analysis performed on cultures that were propagated for at least 5 months, under sterile conditions in a B_12_-free medium, revealed similar levels of the vitamin. Since the results described above were obtained by the microbiological assay method, the reliability of which was recently put in question because lactic bacterium, *L. delbrueckii*, was found to be able to utilize other corrinoids as well [1], we decided to further study the B_12_ nature in the Mankai plant tissue.

The LC-MS/MS method is a reliable method to analyze and identify vitamin B_12_ and its congeneric forms. We analyzed the four major forms of the vitamin: OH-B_12_, Ado-B_12_, Me-B_12_, and CN-B_12_ in all Mankai samples. The results revealed the presence of all four B_12_ forms in the Mankai samples. It is well known that in animal cells, Me-B_12_ serves as a cofactor for methionine synthase, while Ado-B_12_ is a cofactor of methylmalonyl-CoA mutase. However, plants contain no cobalamin-dependent enzymes [31] and therefore, while one can assume that these metabolites do not play a biological role in Mankai plants, it remains possible that the coenzyme forms of B_12_ are produced in endophytic bacteria, which are the presumed source of the B_12_. As the analysis was performed using the KCN extraction method, we were unable to assess the original content of each of the three natural B_12_ forms in Mankai. However, we were able to determine the total level of B_12_ in Mankai, and importantly, these results were comparable to the microbiological assay method. Moreover, we further investigated the presence of pseudo B_12_ due to reports on the identification of large quantities of this compound in non-animal food sources, such as algae [27]. Since pseudo CN-B_12_ is not commercially available, we used spirulina extracts as a reference source of pseudo CN-B_12_ and compared it with Mankai extracts. Under the LC-MS/MS conditions used in this study, no pseudo CN-B_12_ forms bearing identity with the pseudo CN-B_12_ seen in the spirulina extract were detected in any of the Mankai samples. Therefore, the bioassay analysis is a reliable method to measure vitamin B12 levels in Mankai.

Although the affinity of the gastric intrinsic factor binding protein for authentic B_12_ is 500 times greater than for pseudo B_12_ [32,33], according to Herbert and Drivas [34], non-cobalamin vitamin B_12_ analogues, produced by algae and other organisms, may interfere with vitamin B_12_ metabolism. A recent study by Bito et al. demonstrated that pseudo B_12_ can inhibit transcobalamin II-mediated absorption in mammalian cultured COS-7 cells [35].

Functional microbial composition analysis based on genome prediction and sequence matching of microbes in reactors that were inoculated with human fecal samples indicated that Mankai-supplemented reactors displayed a significantly enhanced relative abundance of 16S rRNA gene sequences of microorganisms that have the gene required to produce the vitamin B_12_ transporter BtuB. BtuB, located in the outer membrane of Gram-negative bacteria, is essential for the active uptake of cobalamin across the outer membrane [36]. We could infer that the increased abundance in gut microorganisms that produce the vitamin B_12_ transporter is due to the increased abundance of thisvitamin B_12_ in the Mankai reactors. Vitamin B_12_ is an essential cofactor in several microbial anaerobic processes (e.g., propionate fermentation, butyrate fermentation via 3-methylaspartate, methanogenesis), suggesting that this vitamin has the potential to stimulate fermentation and, thus, the production of short-chain fatty acids [37,38,39,40,41], which provide many benefits to the host [42].

The origin of the vitamin B_12_ in Mankai was not determined in this study, but we speculate that it is derived from an endophyte bacterial source. The fact that we did find B_12_ in the axenic cultures does not negate this hypothesis as axenic duckweed cultures, although often termed in the literature as “sterile” cultures, may still contain a plant tissue that carries microbes, in its internal core, as described by Gilbert et al. [12]. One may reasonably assume that a single or several such endophytic bacteria are responsible for the production of B_12_ found in Mankai.

Collectively, these results indicate that the presence of B_12_ in Mankai is not an occasional event nor a result of uptake from the surrounding medium but is stably and consistently produced within or in close association with the plant. Further studies should be conducted to identify the vitamin B_12_-producing bacteria and characterize their interaction with the plant. These studies may lead to novel strategies for B_12_ enrichment in Mankai and would contribute to its nutritional value as a potential vitamin B_12_ food source, particularly for individuals who prefer a vegetarian lifestyle or who eschew any animal products in their diet.

The recommended dietary allowance of vitamin B_12_ for adults is set at 2.4 μg/day [43]. The vitamin B_12_ content in Mankai, according to our repeated analyses, is about 0.5 μg/20 g DW (equivalent to 100 g of frozen Mankai, as given to our participants as a green dinner shake), thus making it a desirable plant substitute. Although advised to completely reduce red meat intake, we observed a significant increase in vitamin B_12_ levels among participants who were under a semi-vegetarian weight loss diet, compared with participants who, although advised to adopt a healthy lifestyle, did not significantly change their routine red meat intake. It has to be noted that a significant trend in vitamin B_12_ increase was observed between the intervention groups, even though the green-MED dieters were instructed to avoid red/processed meat and their diet was further fortified with Mankai shake and green tea. In addition, participants who reduced red meat had an increase in serum folate (a marker for green leafy vegetable consumption [22]), and in this study for Mankai consumption [17] had an increase in vitamin B_12_ comparable to participants who increased red meat and had a decrease in serum folate levels. Reducing red meat consumption, especially processed meat products, has been a focus of attention in recent years, due to increasing evidence of the association between meat consumption and health risks [44]. However, reducing red meat, as vegan or some vegetarian eating patterns suggest, might put one at risk of vitamin B_12_ deficiency, which could result in megaloblastic anemia and neurological damage [45,46]. Vegetarians and vegans in particular are at risk of developing vitamin B_12_ deficiency and infants born to mothers who follow such diets run a risk of neurodevelopmental abnormalities and feeding difficulties [47]. Therefore, the identification of a natural alternative vitamin B_12_ source would be of major interest to nutrition professionals.

Natural sources of authentic vitamin B_12_ include red meat and fish but also dairy and eggs [31,46]. However, it is well known that growing cattle for food requires a lot of land, water, and energy, and generates considerable waste [48,49]. In the search for a sustainable vitamin B_12_ source, it has been reported that some plant foods (e.g., mushrooms and edible Algae) are rich in corrinoids, but those foods either lack the bioactive form of vitamin B_12_, must be consumed in impractical amounts, or because of controversial data are a questionable source of bioavailable B_12_ [50,51,52]. Alternatively, insects have been proposed as a promising source of food for vitamin B_12_. Mealworms, grasshoppers, crickets, and cockroaches were studied regarding their content of bioactive vitamin B_12_ but exhibited marked variations in their vitamin B_12_ content [53]. Moreover, esthetic, religious, and psychological barriers may further limit their use as a source of vitamin B_12_ replacement.

The limitations of this study include the inability to assess the origin of the vitamin B_12_ in the plant, as well as the bioavailability and specific digestibility pathway of vitamin B_12_ directly among our human participants. The bioassay method, based on the B_12_-requiring bacteria *Lactobacillus Delbrueckii*, cannot determine whether Mankai contains cobalamin or inactive corrinoids or both [1]. However, the fact that the LC-MS/MS method, which is a direct physico-chemical assay for B_12_, revealed comparable levels to the bioassay method indicates that, in the case of Mankai, the bioassay results reflect solely the concentrations of authentic B_12_ forms and not analogues. We were also not able to isolate Mankai as a sole source of vitamin B_12_ from other dietary components rich in vitamin B_12_ in the long-term human trial. In order to overcome this limitation, we presented additional analyses from the electronic questionnaires of other B_12_ sources. We did not measure homocysteine or methylmalonic acid, which might better reflect metabolic deficiencies of vitamin B_12_ or serum folate [54], thus we cannot evaluate the effect of the intervention in cases with low B_12_ and high levels of these serum/plasma markers indicative of biochemical B_12_ deficiency. Furthermore, our participants had baseline serum B_12_ levels within the normal range, so, although we could observe significant increases, we could not demonstrate efficacy for correction of B_12_ deficiency status and further studies should be carried out to examine this question. We also cannot point out the exact mechanism that explains the substantial B_12_ content in the Mankai plant, nor the way in which this may be controlled in the plant tissue. The data we showed for our bioreactors are from a small pilot study, and we consider them preliminary. Thus, an open question remains concerning the possibility that the Mankai plant may modify the microbiota in the intestinal tract with possible effects on the bioavailability of B_12_ normally present in bile [5]. Strengths of the data that we report here include the comprehensive multi-assessment of several aspects of B_12_, including laboratory, gut-related, and a long-term human randomized controlled trial, with monitored lunch and daily supply of Mankai to the participants.

## 5. Conclusions

The Mankai plant contains bioactive B_12_ compounds and could potentially serve as a plant-based food source of vitamin B_12_. Results from this study could provide additional insight regarding a much-needed alternative healthy and sustainable B_12_ source.

## Figures and Tables

**Table 1:** Baseline characteristics of the DIRECT PLUS participants across sex-specific vitamin B_12_ tertiles.

	Entire *n* = 294	Lowest Tertile *n* = 99	Intermediate Tertile *n* = 98	Highest Tertile *n* = 97	*p* Between Tertiles ^1^	*p* Between Extreme Tertiles ^2^
Vitamin B_12_, pg/mL	420.4 ± 187	261.2 ± 46.1	385.7 ± 37.9	618.1 ±1 92.8	***-***	***-***
Age, years	51.1 ± 10.5	51.9 ± 9.6	49.7 ± 10.5	51.5 ± 11.4	0.25	0.54
Men, number	259	87	86	86	0.98	-
BMI, kg/m^2^	31.3 ± 4.0	31.3 ± 4.3	31.3 ± 3.9	31.2 ± 3.7	0.84	0.87
WC, cm	109.7 ± 9.5	110.1 ± 9.7	109.6 ± 10.5	109.4 ± 8.1	0.67	0.93
Fasting glucose, mg/dL	101.9 ± 17.1	104.0 ± 19.5	101.2 ± 14.7	100.6 ± 16.5	0.62	0.35
Cholesterol, mg/dL	190.6 ± 33.0	190.9 ± 29.5	190.9 ± 33.8	189.8 ± 35.9	0.97	0.82
HDL-c, mg/dL	46.0 ± 11.7	45.0 ± 12.4	46.2 ± 11.3	46.7 ± 11.3	0.29	0.15
LDL-c, mg/dL	125.7 ± 30.1	125.5 ± 28.6	127.0 ± 31.6	124.5 ± 32.4	0.86	0.83
Triglycerides, mg/dL	146.3 ± 66.8	159.4 ± 66.9	139.8 ± 60.0	139.5 ± 68.8	0.02	0.01
ALT, U/L	34.9 ± 16.8	34.3 ± 14.4	35.4 ± 20.4	34.9 ± 15.0	0.79	0.71
AST, U/L	25.6 ± 7.7	25.5 ± 7.2	26.1 ± 8.7	25.3 ± 7.3	0.90	0.74

Continuous data presented as means ± SD. Lowest tertile: Men: < = 322.49 pg/mL; Women: < = 318.43 pg/mL. Intermediate tertile: Men: 322.50 pg/mL–439.02 pg/mL; Women: 318.44 pg/mL–478.32 pg/mL. Highest tertile: Men: 439.03 + pg/mL; women: 478.33 + pg/mL. ^1^ tested using ANOVA/Kruskal-Wallis. ^2^ tested using *T*-test/Mann-Whitney. ALT, alanine transaminase; AST, aspartate transaminase. BMI, body mass index; HDL-c, high density lipoprotein cholesterol; LDL-c, low density lipoprotein cholesterol; WC, waist circumference.

## Supplementary Materials

The following are available: https://www.mdpi.com/2072-6643/12/10/3067/s1. File S1: Further details on the extraction and purification of Vitamin B_12_, File S2: Exclusion criteria DIRECT PLUS trial, File S3: Further outcome measurements of DIRECT PLUS trial, File S4: Full details regarding the media, anoxic bioreactor, Mankai lysate and the sampling, File S5: Further details regarding the 16S rRNA amplicon sequences, File S6: Supplementation usage, DIRECT PLUS trial, Table S1: LC and MS parameters for detection of corrinoids, Table S2: Composition of micronutrient-containing solution and macronutrient solution that were used to prepare Base medium and Mankai medium, Table S3: Relative 16S rRNA gene amplicon abundance and taxonomy of phylotypes that (a) are predicted to contain btuB in their genome and (b) displayed a greater than 0.5% relative abundance in either the Mankai-supplemented or control reactor, Figure S1: The DIRECT PLUS trial flow diagram, Figure S2: Comparison of chromatograms of different MRMs for CN-B_12_ standard 0.1 µg/mL (A-D) and plant sample (E-H), Figure S3: Liquid chromatography/electrospray ionization−tandem mass spectrometry chromatograms of bioactive B_12_ compounds, Figure S4: Comparison of chromatograms of different MRMs for Pseudo CN-B12 in Mankai (A-D) and Spirulina (E-H) samples, Figure S5: Further nutritional analysis.

## References

[1] Watanabe F., Bito T. (2018). Determination of cobalamin and related compounds in foods. J. AOAC Int..

[2] Burgess C.M., Smid E.J., van Sinderen D. (2009). Bacterial vitamin B2, B11 and B12 overproduction: An overview. Int. J. Food Microbiol..

[3] Green R. (2017). Vitamin B12 deficiency from the perspective of a practicing hematologist. Blood J. Am. Soc. Hematol..

[4] Watanabe F., Takenaka S., Kittaka-Katsura H., Ebara S., Miyamoto E. (2002). Characterization and bioavailability of vitamin B12-compounds from edible algae. J. Nutr. Sci. Vitaminol..

[5] Green R., Miller J.W., Zempleni J., Suttie J.W., Gregory J.F., Stover P.J. (2013). Vitamin B12. Handbook of Vitamins.

[6] Rizzo G., Laganà A.S., Rapisarda A.M.C., Ferrera L., Grazia G.M., Buscema M., Rossetti P., Nigro A., Muscia V., Valenti G. (2016). Vitamin B12 among vegetarians: Status, assessment and supplementation. Nutrients.

[7] Crowe F.L., Appleby P.N., Travis R.C., Key T.J. (2013). Risk of hospitalization or death from ischemic heart disease among British vegetarians and nonvegetarians: Results from the EPIC-Oxford cohort study. Am. J. Clin. Nutr..

[8] Yokoyama Y., Barnard N.D., Levin S.M., Watanabe M. (2014). Vegetarian diets and glycemic control in diabetes: A systematic review and meta-analysis. Cardiovasc. Diagn. Ther..

[9] Wang F., Zheng J., Yang B., Jiang J., Fu Y., Li D. (2015). Effects of vegetarian diets on blood lipids: A systematic review and meta-analysis of randomized controlled trials. J. Am. Heart Assoc..

[10] Yokoyama Y., Nishimura K., Barnard N.D., Miyamoto Y. (2017). Blood pressure and vegetarian diets. Vegetarian and Plant-Based Diets in Health and Disease Prevention.

[11] Sukumar N., Saravanan P. (2019). Investigating vitamin B12 deficiency. BMJ.

[12] Gilbert S., Xu J., Acosta K., Poulev A., Lebeis S., Lam E. (2018). Bacterial production of indole related compounds reveals their role in association between duckweeds and endophytes. Front. Chem..

[13] Appenroth K.J., Sree K.S., Bog M., Ecker J., Boehm V., Lorkowski S., Sommer K., Vetter W., Tolzin-Banasch K., Kirmse R. (2018). Nutritional value of the duckweed species of the genus Wolffia (*Lemnaceae*) as human food. Front. Chem..

[14] Kawamata Y., Shibui Y., Takumi A., Seki T., Shimada T., Hashimoto M., Inoue N., Kobayashi H., Narita T. (2020). Genotoxicity and repeated-dose toxicity evaluation of dried Wolffia globosa Mankai. Toxicol. Rep..

[15] Kaplan A., Zelicha H., Tsaban G., Yaskolka Meir A., Rinott E., Kovsan J., Novack L., Thiery J., Ceglarek U., Burkhardt R. (2019). Protein bioavailability of Wolffia globosa duckweed, a novel aquatic plant–A randomized controlled trial. Clin. Nutr..

[16] Yan Y., Candreva J., Shi H., Ernst E., Martienssen R., Schwender J., Shanklin J. (2013). Survey of the total fatty acid and triacylglycerol composition and content of 30 duckweed species and cloning of a Δ6-desaturase responsible for the production of γ-linolenic and stearidonic acids in Lemna gibba. BMC Plant Biol..

[17] Yaskolka Meir A., Tsaban G., Zelicha H., Rinott E., Kaplan A., Youngster I., Rudich A., Shelef I., Tirosh A., Brikner D. (2019). A Green-mediterranean diet, supplemented with mankai duckweed, preserves iron-homeostasis in humans and is efficient in reversal of anemia in rats. J. Nutr..

[18] Ball G.F.M. (1994). Microbiological methods for the determination of the B-group vitamins. Water-Soluble Vitamin Assays in Human Nutrition.

[19] Rinott E., Youngster I., Meir A.Y., Tsaban G., Zelicha H., Kaplan A., Knights D., Tuohy K., Fava F., Scholz M.U. (2020). Effects of diet-modulated autologous fecal microbiota transplantation on weight regain. Gastroenterology.

[20] Shai I., Schwarzfuchs D., Henkin Y., Shahar D.R., Witkow S., Greenberg I., Golan R., Fraser D., Bolotin A., Vardi H. (2008). Weight loss with a low-carbohydrate, Mediterranean, or low-fat diet. N. Engl. J. Med..

[21] Gepner Y., Shelef I., Schwarzfuchs D., Zelicha H., Tene L., Meir A.Y., Tsaban G., Cohen N., Bril N., Rein M. (2018). Effect of distinct lifestyle interventions on mobilization of fat storage pools: Central magnetic resonance imaging randomized controlled trial. Circulation.

[22] Moll R., Davis B. (2017). Iron, vitamin B12 and folate. Medicine.

[23] McDonald J.A.K., Schroeter K., Fuentes S., Heikamp-deJong I., Khursigara C.M., de Vos W.M., Allen-Vercoe E. (2013). Evaluation of microbial community reproducibility, stability and composition in a human distal gut chemostat model. J. Microbiol. Methods.

[24] Gutierrez D., Weinstock A., Antharam V.C., Gu H., Jasbi P., Shi X., Dirks B., Krajmalnik-Brown R., Maldonado J., Guinan J. (2020). Antibiotic-induced gut metabolome and microbiome alterations increase the susceptibility to Candida albicans colonization in the gastrointestinal tract. FEMS Microbiol. Ecol..

[25] Ilhan Z.E., di Baise J.K., Dautel S.E., Isern N.G., Kim Y.M., Hoyt D.W., Schepmoes A.A., Brewer H.M., Weitz K.K., Metz T.O. (2020). Temporospatial shifts in the human gut microbiome and metabolome after gastric bypass surgery. NPJ Biofilms Microbiomes.

[26] Jägerstad M., Arkbåge K., Caballero B., Trugo L.C., Finglas P.M. (2003). Cobalamins properties and determination. Encyclopedia of Food Sciences and Nutrition.

[27] Watanabe F., Katsura H., Takenaka S., Fujita T., Abe K., Tamura Y., Nakatsuka T., Nakano Y. (1999). Pseudovitamin B12 is the predominant cobamide of an algal health food, spirulina tablets. J. Agric. Food Chem..

[28] Chamlagain B., Edelmann M., Kariluoto S., Ollilainen V., Piironen V. (2015). Ultra-high performance liquid chromatographic and mass spectrometric analysis of active vitamin B12 in cells of Propionibacterium and fermented cereal matrices. Food Chem..

[29] Langille M.G.I., Zaneveld J., Caporaso J.G., McDonald D., Knights D., Reyes J.A., Clemente J.C., Burkepile D.E., Thurber R.L.V., Knight R. (2013). Predictive functional profiling of microbial communities using 16S rRNA marker gene sequences. Nat. Biotechnol..

[30] Watanabe F., Yabuta Y., Bito T., Teng F. (2014). Vitamin B12-containing plant food sources for vegetarians. Nutrients.

[31] Watanabe F. (2007). Vitamin B12 sources and bioavailability. Exp. Biol. Med..

[32] Fedosov S.N., Fedosova N.U., Kräutler B., Nexø E., Petersen T.E. (2007). Mechanisms of discrimination between cobalamins and their natural analogues during their binding to the specific B12-transporting proteins. Biochemistry.

[33] Stupperich E., Nexø E. (1991). Effect of the cobalt-N coordination on the cobamide recognition by the human vitamin B12 binding proteins intrinsic factor, transcobalamin and haptocorrin. Eur. J. Biochem..

[34] Herbert V., Drivas G. (1982). Spirulina and vitamin B12. JAMA.

[35] Bito T., Bito M., Hirooka T., Okamoto N., Harada N., Yamaji R., Nakano Y., Inui H., Watanabe F. (2020). Biological activity of pseudovitamin B12 on cobalamin-dependent methylmalonyl-CoA mutase and methionine synthase in mammalian cultured COS-7 Cells. Molecules.

[36] Schauer K., Rodionov D.A., de Reuse H. (2008). New substrates for TonB-dependent transport: Do we only see the ‘tip of the iceberg’?. Trends Biochem. Sci..

[37] Xu Y., Xiang S., Ye K., Zheng Y., Feng X., Zhu X., Chen J., Chen Y. (2018). Cobalamin (vitamin B12) induced a shift in microbial composition and metabolic activity in an in vitro colon simulation. Front. Microbiol..

[38] Louis P., Flint H.J. (2017). Formation of propionate and butyrate by the human colonic microbiota. Environ. Microbiol..

[39] Takahashi-Iñiguez T., García-Hernandez E., Arreguín-Espinosa R., Flores M.E. (2012). Role of vitamin B12 on methylmalonyl-CoA mutase activity. J. Zhejiang Univ. Sci. B.

[40] Banerjee R., Ragsdale S.W. (2003). The many faces of vitamin B12: Catalysis by cobalamin-dependent enzymes. Annu. Rev. Biochem..

[41] Buckel W. (2001). Unusual enzymes involved in five pathways of glutamate fermentation. Appl. Microbiol. Biotechnol..

[42] Van Treuren W., Dodd D. (2020). Microbial contribution to the human Metabolome: Implications for health and disease. Annu. Rev. Pathol. Mech. Dis..

[43] Food and Nutrition Board, Institute of Medicine (2004). Dietary Reference Intakes: Recommended Intakes for Individuals.

[44] Micha R., Penalvo J.L., Cudhea F., Imamura F., Rehm C.D., Mozaffarian D. (2017). association between dietary factors and mortality from heart disease, stroke, and Type 2 diabetes in the united states. JAMA.

[45] Obeid R., Heil S.G., Verhoeven M.M.A., van den Heuvel E.G.H.M., de Groot L.C., Eussen S.J.P.M. (2019). Vitamin B12 intake from animal foods, biomarkers, and health aspects. Front. Nutr..

[46] Green R., Allen L.H., Bjørke-Monsen A.L., Brito A., Guéant J.L., Miller J.W., Molloy A.M., Nexo E., Stabler S., Toh B.H. (2017). Vitamin B 12 deficiency. Nat. Rev. Dis. Prim..

[47] Oussalah A., Levy J., Berthezène C., Alpers D.H., Guéant J.L. (2020). Health outcomes associated with vegetarian diets: An umbrella review of systematic reviews and meta-analyses. Clin. Nutr..

[48] Lebacq T., Baret P.V., Stilmant D. (2013). Sustainability indicators for livestock farming. A review. Agron. Sustain. Dev..

[49] Van Zanten H.H.E., Herrero M., van Hal O., Röös E., Muller A., Garnett T., Gerber P.J., Schader C., Boer I.J.M. (2018). De Defining a land boundary for sustainable livestock consumption. Glob. Chang. Biol..

[50] Watanabe F., Yabuta Y., Tanioka Y., Bito T. (2013). Biologically active vitamin B12 compounds in foods for preventing deficiency among vegetarians and elderly subjects. J. Agric. Food Chem..

[51] Suzuki H. (1995). Serum vitamin B12 levels in young vegans who eat brown rice. J. Nutr. Sci. Vitaminol..

[52] Dagnelie P.C., van Staveren W.A., van den Berg H. (1991). Vitamin B-12 from algae appears not to be bioavailable. Am. J. Clin. Nutr..

[53] Schmidt A., Call L.M., Macheiner L., Mayer H.K. (2019). Determination of vitamin B12 in four edible insect species by immunoaffinity and ultra-high performance liquid chromatography. Food Chem..

[54] Klee G.G. (2000). Cobalamin and folate evaluation: Measurement of methylmalonic acid and homocysteine vs vitamin B12 and folate. Clin. Chem..

