# Supplementary material for: Wolffia globosa–Mankai Plant-Based Protein Contains Bioactive Vitamin B12 and Is Well Absorbed in Humans
Source: Nutrients. 2020 Oct 8;12(10):3067. doi: 10.3390/nu12103067 (PMC7600829; doi:10.3390/nu12103067)
Supplement: Supplementary file 1 [file nutrients-12-03067-s001.pdf]

Supplementary material

Supplementary File S1: Further details on the extraction and analysis of Vitamin B_{12}

Extraction of Vitamin B_{12}: Total vitamin B_{12} was extracted from the homogenates by boiling with KCN at acidic pH [1] as follows: dry sample (1g) was fine powdered using a mortar and pestle, transferred to 20mL of 0.5M acetate buffer, pH 4.5, and homogenized with a vortex for 30 seconds. 250µl of 1% KCN solution and 300mg of α-amylase (Sigma-Aldrich, Switzerland) were added to the sample solutions and incubated for 1h at 37°C. Sample volume was adjusted to 40ml and homogenates were incubated at 95°C for 30 min. After cooling, the tubes were centrifuged at 10,000 rpm for 10 min, and the collected supernatants were filtered through 0.45 µm nylon membrane and kept at 4°C, until use. All procedures were done in the dark

LC-MS/MS analysis: The procedure was performed on an instrument consisting of Acquity I-class UPLC and Xevo TQ-S systems (both Waters, Milford, MA). The separation was performed on BEH C18 column (1.7um, 2.1 x 50 mm, Waters) using a linear gradient of methanol (solvent B) in 10µM ammonium formate, pH 3.5 (solvent A) as follows: %B (time interval, min): 0 (0-0.2), 0-35 (0.02-1.5), 35-98 (1.5-2), 98 (2-4), 98-0 (4-4.2), 0 (4.2-7), at 25°C and flow 0.3 mL/min. The electrospray ionization−mass spectroscopy (ESI-MS) system was operated in the positive ion mode, with capillary voltage of 3.07 kV, cone voltage 37 V, desolvation gas 700 L/h, cone gas 150 L/h, and argon as collision gas (0.10 mL/min). Source and desolvation temperatures were 120°C and 400°C, respectively.

Supplementary File S2: Exclusion criteria DIRECT PLUS trial

Inability to partake in physical activity (PA), a serum creatinine level≥2mg/dL, disturbed liver function, major illness that might require hospitalization, pregnancy or lactation for women, presence of active cancer or undergoing chemotherapy either at present or in the prior three years, participation in another trial, chronic treatment with warfarin (given its interaction with vitamin K), and being implanted with a pacemaker or platinum implant (due to inability to undergo magnetic resonance imaging included in the study design).

Supplementary File S3: Further outcome measurements of DIRECT PLUS trial

Anthropometric: Height was measured to the nearest millimeter using a standard wall-mounted stadiometer. Body weight was measured without shoes to the nearest 0.1 kg. WC was measured halfway between the last rib and the iliac crest to the nearest millimeter by standard procedures using an anthropometric measuring tape.

Electronic questionnaires: Food frequency electronic questionnaires were administered at baseline and at the end of the trial [2,3]. We followed overall changes in specific food groups' intake, as

described previously [4] and further used lifestyle and validated PA questionnaire [5]. The intake green Mankai shake was monitored using specific questions included in the electronic questionnaires for the evaluation of the amount of intake.

Supplementary File S4: Full details regarding the media, anoxic bioreactor, Mankai lysate and the sampling

Two anoxic media were used to examine the potential effect of Mankai on human-derived gut microbiota. Both media were based on the protocol described by McDonald et al [6], with the following modification in order to provide the same Chemical Oxygen Demand (COD) amount (200meq/L) to all treatments. The final media consisted of an anoxic micronutrient-containing solution and an anoxic macronutrient solution (**Table S2**). COD was measured to quantify the reducing equivalents in both solutions.

To obtain a (a) Base medium for the bioreactors that lacked Mankai and for the starter culture (see above), and (b) Mankai medium for the Mankai-supplemented bioreactors, micronutrient-containing solution and macronutrient solution were combined, accordingly (**Table S2**). Reducing equivalents of both media were quantified again by measuring the COD. Three 35 mL serum bottles were either filled with 20 mL of Base medium or 18.8 mL of Mankai medium (NOTE: six bioreactors in total). Remaining volume was used for fill-and-draw. All medium-containing bottles were autoclaved and stored at room temperature.

Before bioreactor inoculation (adding 1ml of the starter culture [see above]), Mankai lysate was prepared by blending 5g of frozen Mankai (*Wolffia globosa* var. Mankai) with 400mL of DI water for five minutes and subsequently flushing with nitrogen for 5min. 1.2mL of the Mankai lysate (containing 20 reducing equivalent) was added to each of the three Mankai medium-containing bioreactors. The remaining Mankai lysate was stored at -20°C with an anoxic sachet (BD GasPak EZ Pouch) for fill-and-draw. After inoculation and before the first fill-and-draw, the bioreactors were incubated for 48h in the dark at 37°C and mixed continuously at 100rpm. Fill-and-draw was repeated every 48h until the end of the incubation period at day 14. 10mL (11 ml at first fill-and-draw) were removed and replaced by either (a) 10mL anoxic autoclaved Base medium [Control Reactors] or (b) 8.8mL anoxic autoclaved Mankai medium and 1.2ml thawed Mankai lysate [Mankai Reactors]. Between fill-and-draw steps, reactors were incubated in a shaking incubator at 37°C in the dark. 2 mL of each sample obtained from fill-and-draw cycles was centrifuged at 13200rpm for 5 min and pellets were stored at -80°C until DNA extraction.

Supplementary File S5: Further detailing regarding the 16S rRNA amplicon sequences

Using the 16S rRNA gene amplicon sequences, we match to available genomes and predicted possible microbial functions using Phylogenetic Investigation of Communities by Reconstruction of Unobserved States PICRUSt [7], finally we performed Linear discriminant analysis Effect Size LEfSe as described [8], to find statistically significant difference in microbial enzymes and pathways identified.

Supplementary File S6: Supplementation usage, DIRECT PLUS trial

At baseline, a total of 13 participants reported on multivitamin usage (p=0.22 between intervention groups), 18 participants reported any Vitamin B supplement (p=0.45 between intervention groups), and 9 participants reported on specific vitamin B_{12} supplement (p=0.05 between groups, with the following distribution: HDG=3 participants; Green-MED/low meat had 6 participants reporting B_{12} supplement).

At the end of the intervention, vitamin supplementation usage did not differ between the intervention groups, and was similar to the distribution reported at baseline (multivitamin usage: p=0.83; any of B group vitamins: p=0.5; supplementation of vitamin B_{12}: p=0.09).

Table S1: LC and MS parameters for detection of corrinoids:

Compound	Parent ion, m/z	Daughter ions (collision), m/z (V)	Retention time, min
CN-B_{12}	678.4	147.1(45), 359.0(25), 912.3(35), 997.3(25)	2.11
OH-B_{12}	664.8	147.2(37), 635.8(17), 912.4(27)	1.87
Ade-B_{12}	790.4	147.3(52), 665.5(27)	2.25
Me-B_{12}	673.0	147.2(42), 359.0(27), 665.2(17), 971.4(27)	2.31
Pseudo CN-B_{12}	672.5*	136.0(45), 348.0(25), 912.0(35), 997.0(25)*	2.09

* In the absence of Pseudo CN-B_{12} standard we assumed its fragmentation pattern similar to that of CN-B12, with a corresponding correction for molecular masses.

Table S2: Composition of micronutrient-containing solution and macronutrient solution that were used to prepare Base medium and Mankai medium

Medium	Micronutrient-containing Solution (500 mL)			Macronutrient Solution (2 L)	
	**Weight (g)**	**Reagent**		**Weight (g)**	**Reagent**
	0.02	CaCl		0.4	Peptone
	0.2	NaCl		0.4	Yeast Extract
	0.08	K_2HPO_4		0.4	Pectin
	0.08	KH_2PO_4		1.0	Potato Starch
	0.02	$MgSO_4$		0.4	Arabinogalactan
	8.0	$NaHCO_3$		0.6	Casein
	0.002	Menadione		0.2	Inulin
	2.0	Cysteine		0.8	Mucin
	0.01	Hemine		0.1	Bile Salts
**Base**[a]		250 mL	+		734.80 mL
**Mankai**[b]		250 mL	+		504.67 mL

[a] 200 meq/L; DI water was added to a total volume of 1 L

[b] 180 meq/L; DI water was added to a total volume of 940 L

Table S3: Relative 16S rRNA gene amplicon abundance and taxonomy of phylotypes that (a) are predicted to contain btuB in their genome and (b) displayed a greater than 0.5% relative abundance in either the Mankai-supplemented or control reactor.

Phylotype	Mankai	Control	Order	Family	Species	Identity (%)
PT_27*	4.2 ± 0.3	0.0 ± 0.0	*Aeromonadales*	*Aeromonadaceae*	*Aeromonas hydrophila*	100
PT_20*	3.2 ± 1.3	0.0 ± 0.0	*Burkholderiales*	*Comamonadaceae*	*Pelomonas aquatica, Pelomonas puraquae*	100
PT_2	1.6 ± 1.6	0.0 ± 0.0	*Xanthomonadales*	*Xanthomonadaceae*	*Stenotrophomonas maltophilia*	100
PT_49	1.6 ± 2.4	0.0 ± 0.0	*Enterobacteriales*	*Enterobacteriaceae*	*Citrobacter freundii, Raoultella terrigena, Citrobacter braakii*	100
PT_53*	1.4 ± 0.1	0.0 ± 0.0	*Desulfuromonadales*	*Geobacteraceae*	*Geobacter anodireducens*	100
PT_74	0.7 ± 0.1	0.0 ± 0.0	*Cytophagales*	*Cytophagaceae*	*Runella zeae*	99
PT_73	0.7 ± 0.2	0.0 ± 0.0	*Pseudomonadales*	*Pseudomonadaceae*	*Pseudomonas fulva, Pseudomonas putida*	100
PT_82	0.5 ± 0.2	0.0 ± 0.0	*Burkholderiales*	*Comamonadaceae*	*Comamonas thiooxydans, Comamonas testosteroni*	100
PT_1	0.6 ± 0.2	4.3 ± 0.4	*Enterobacteriales*	*Enterobacteriaceae*	*Escherichia coli*	100

* Phylotypes that were present in all three replicated Mankai-supplemented bioreactors

Figure S1: The DIRECT PLUS trial flow diagram

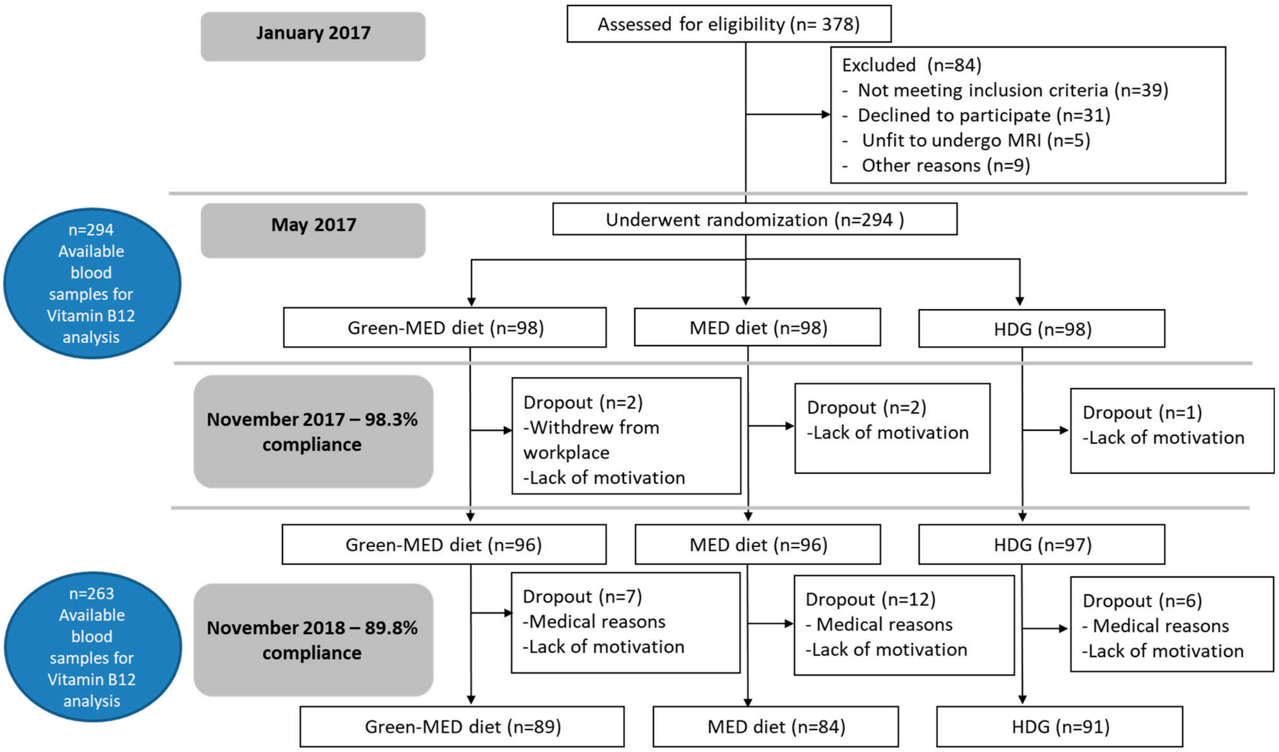

Figure S2: Comparison of chromatograms of different MRMs for CN-B_{12} standard 0.1µg/ml **(A-D)** and plant sample **(E-H)**.

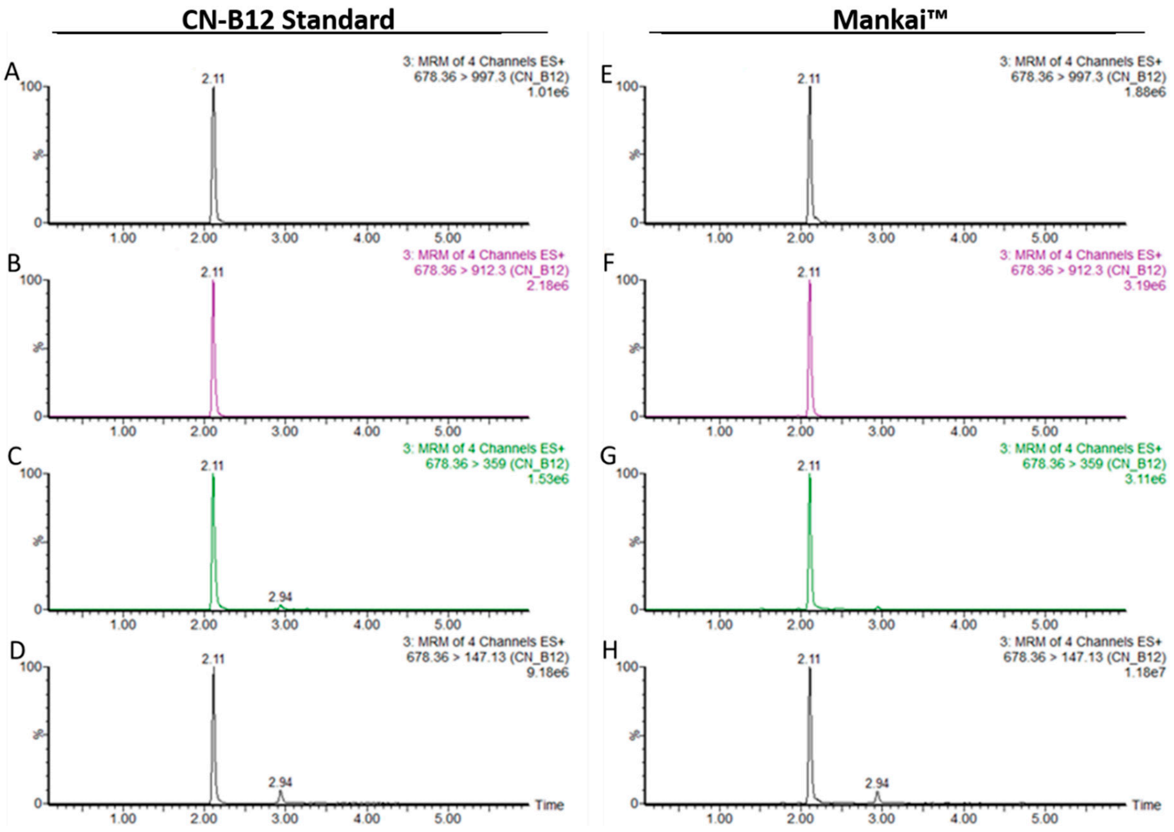

The intensity ratios between individual MRM signals were kept similar in both standard and plant samples.

Figure S3: Liquid chromatography/electrospray ionization–tandem mass spectrometry chromatograms of bioactive B_{12} compounds

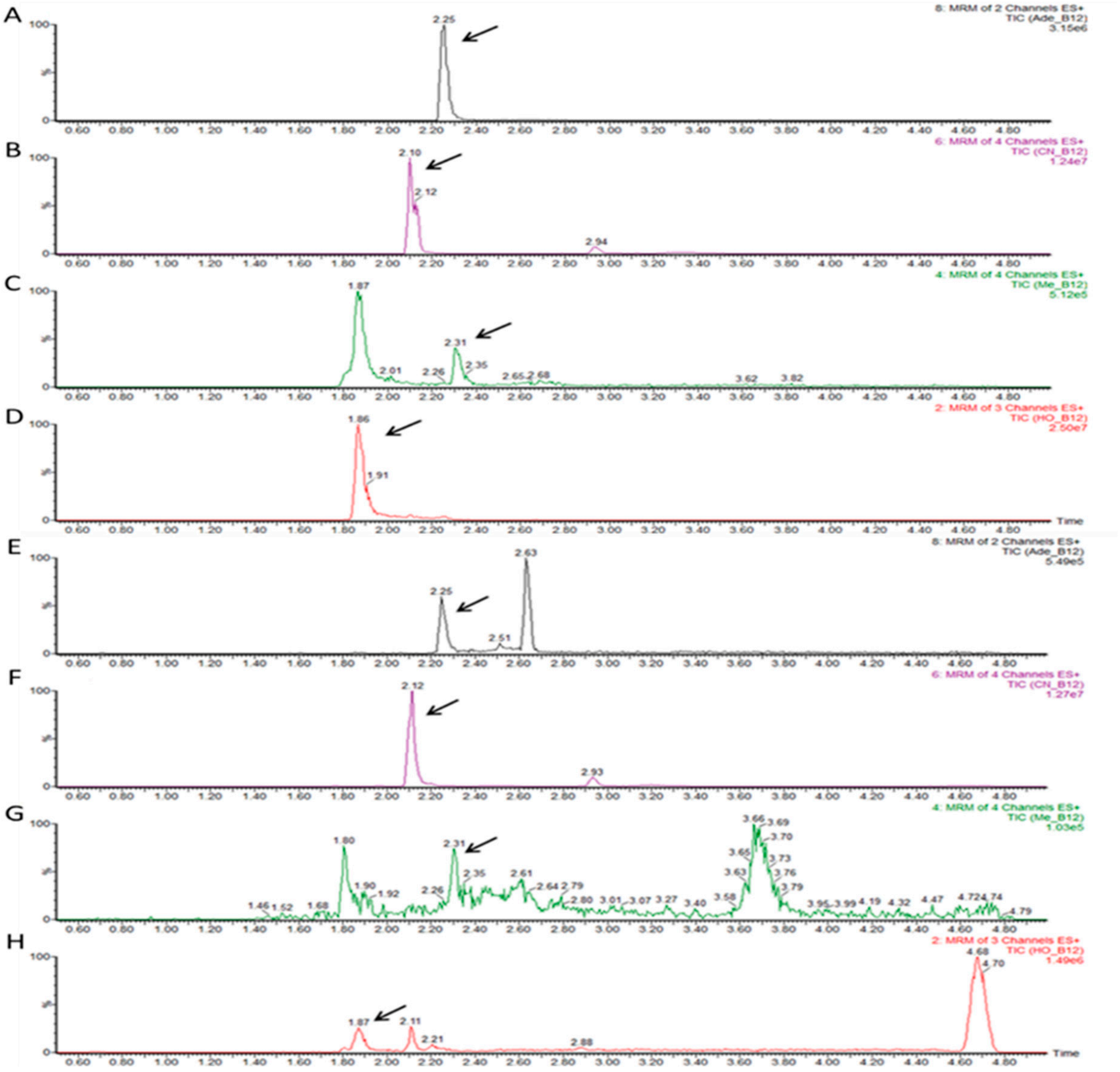

A-D. Commercial standards of Ado-B_{12}, CN-B_{12}, Me-B_{12} and OH-B_{12} (arrows); E-H. The corresponding peaks of B_{12} forms detected in Mankai sample (arrows). The analysis was performed in triplicate.

Figure S4: Comparison of chromatograms of different MRMs for Pseudo CN-B_{12} in Mankai **(A-D)** and Spirulina **(E-H)** samples.

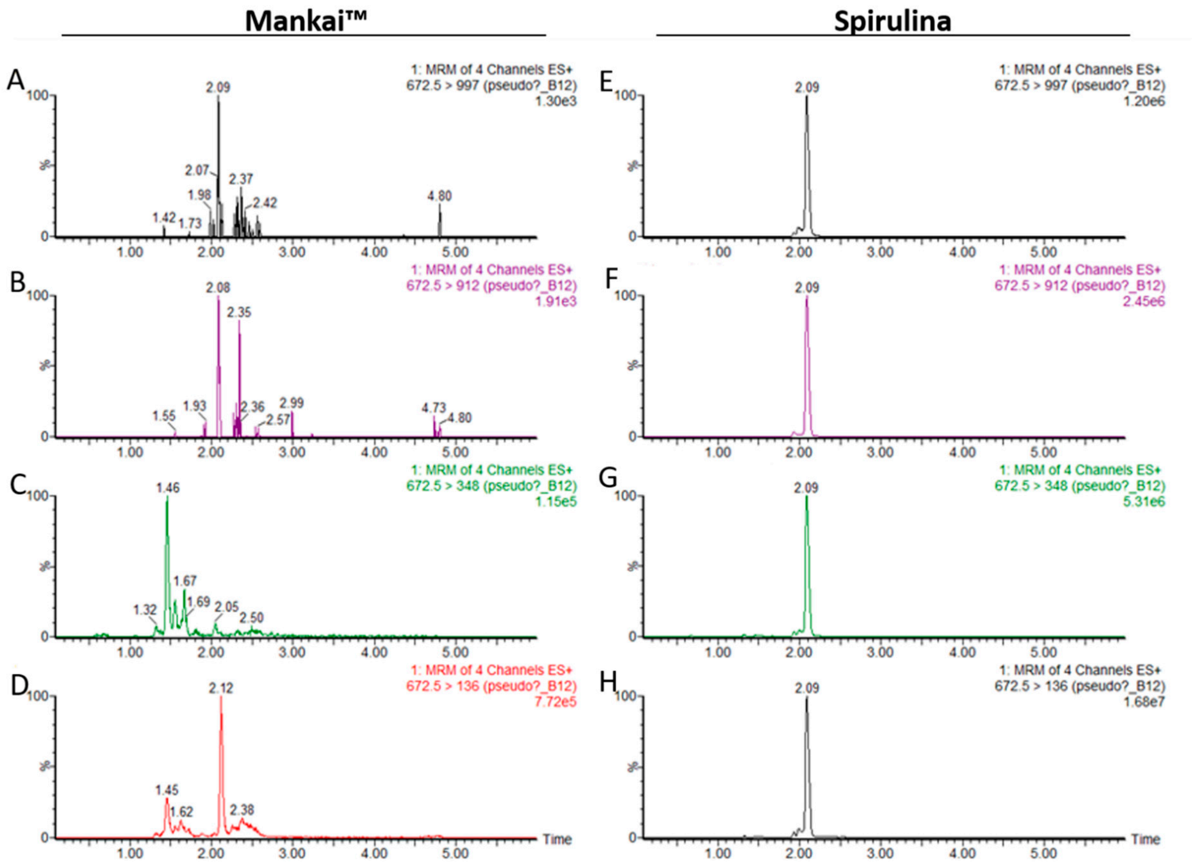

The pseudo CN-B_{12} in Spirulina sample showed strong signals with peak at 2.09 min for all 4 MRM transitions, whereas the signals in Mankai sample, eluted at different retention times and with various intensity in the MRM transitions, implied that pseudo CN-B_{12} is not present in detectable levels.

Figure S5: Further nutritional analysis

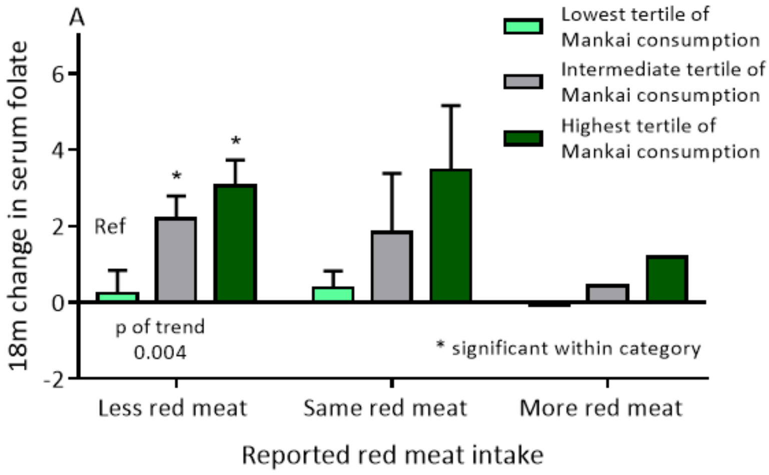

(a) When examining reported red meat consumption (reported as: increased, decreased and no change in consumption) vs. Mankai consumption tertiles, and change in serum folate, marginal differences between groups were observed (p=0.07). Within participants who reported on a decrease in red meat consumption, significant increase in serum folate levels were observed among participants who reported on intermediate and high consumption of Mankai

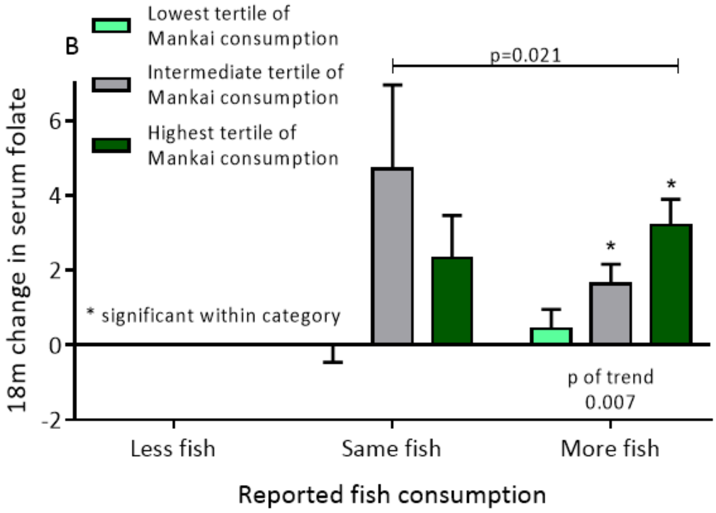

(b) Among participants in group green-MED/low meat, when examining Mankai tertiles vs fish intake change, a significant difference in 18-month change in serum folate between the groups (p=0.007) was observed, as low Mankai consumption/same fish intake was significantly different from high Mankai consumption tertile/more fish (p=0.02). Participants who consumed more Mankai and more Fish, significantly increased their serum folate levels (p<0.05 vs. baseline).

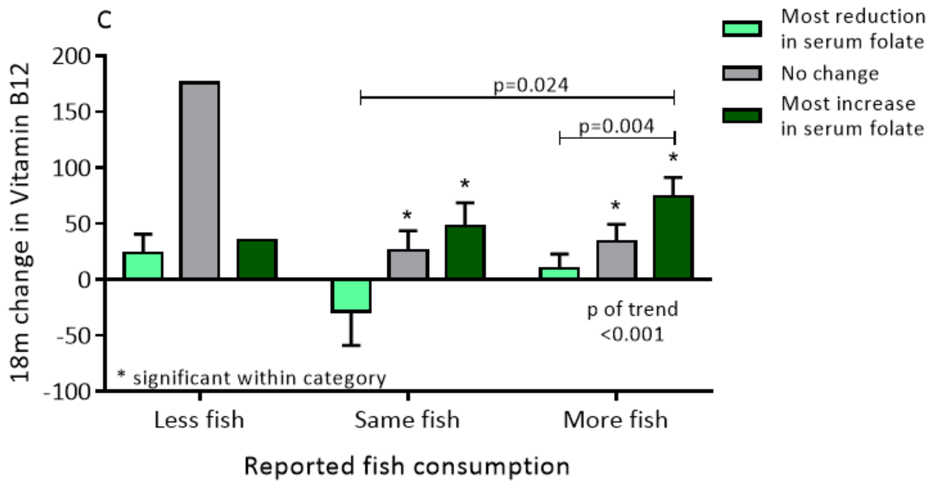

(c) When examining the entire cohort, in fish consumption categories vs. serum folate change (as tertiles), a significant difference was observed between these groups in vitamin B_{12} change (p=0.001). In a specific between group comparisons, reductions in serum folate/same fish was significantly different vitamin B_{12} change, as compared with increased fish/increased levels of serum folate (p=0.024). This group also significantly differed from reduction in serum folate/more fish (p=0.004).

References

1. Frenkel, E.P.; Prough, R.; Kitchens, R.L. [6] Measurement of tissue vitamin B12 by radioisotopic competitive inhibition assay and quantitation of tissue cobalamin fractions. In; Methods in enzymology; Elsevier, 1980; Vol. 67, pp. 31–40.
2. Shai, I.; Shahar, D.R.; Vardi, H.; Fraser, D. Selection of food items for inclusion in a newly developed food-frequency questionnaire. *Public Health Nutr.* **2004**, *7*, 745–749.
3. Shai, I.; Rosner, B.A.; Shahar, D.R.; Vardi, H.; Azrad, A.B.; Kanfi, A.; Schwarzfuchs, D.; Fraser, D. Dietary evaluation and attenuation of relative risk: multiple comparisons between blood and urinary biomarkers, food frequency, and 24-hour recall questionnaires: the DEARR study. *J. Nutr.* **2005**, *135*, 573–579.
4. Gepner, Y.; Golan, R.; Harman-Boehm, I.; Henkin, Y.; Schwarzfuchs, D.; Shelef, I.; Durst, R.; Kovsan, J.; Bolotin, A.; Leitersdorf, E. Effects of initiating moderate alcohol intake on cardiometabolic risk in adults with type 2 diabetes: a 2-year randomized, controlled trial. *Ann. Intern. Med.* **2015**, *163*, 569–579.
5. Chasan-Taber, S.; Rimm, E.B.; Stampfer, M.J.; Spiegelman, D.; Colditz, G.A.; Giovannucci, E.; Ascherio, A.; Willett, W.C. Reproducibility and validity of a self-administered physical activity questionnaire for male health professionals. *Epidemiology* **1996**, 81–86.
6. McDonald, J.A.K.; Schroeter, K.; Fuentes, S.; Heikamp-deJong, I.; Khursigara, C.M.; de Vos, W.M.; Allen-Vercoe, E. Evaluation of microbial community reproducibility, stability and composition in a human distal gut chemostat model. *J. Microbiol. Methods* **2013**, *95*, 167–174.
7. Langille, M.G.I.; Zaneveld, J.; Caporaso, J.G.; McDonald, D.; Knights, D.; Reyes, J.A.; Clemente, J.C.; Burkepile, D.E.; Thurber, R.L.V.; Knight, R. Predictive functional profiling of microbial communities using 16S rRNA marker gene sequences. *Nat. Biotechnol.* **2013**, *31*, 814–821.
8. Segata, N.; Izard, J.; Waldron, L.; Gevers, D.; Miropolsky, L.; Garrett, W.S.; Huttenhower, C. Metagenomic biomarker discovery and explanation. *Genome Biol.* **2011**, *12*, 1–18.
